# Genomic organization and molecular phylogenies of the beta (β) keratin multigene family in the chicken (*Gallus gallus*) and zebra finch (*Taeniopygia guttata*): implications for feather evolution

**DOI:** 10.1186/1471-2148-10-148

**Published:** 2010-05-18

**Authors:** Matthew J Greenwold, Roger H Sawyer

**Affiliations:** 1 Department of Biological Sciences, University of South Carolina, Columbia, SC 29205, USA

## Abstract

**Background:**

The epidermal appendages of reptiles and birds are constructed of beta (β) keratins. The molecular phylogeny of these keratins is important to understanding the evolutionary origin of these appendages, especially feathers. Knowing that the crocodilian β-keratin genes are closely related to those of birds, the published genomes of the chicken and zebra finch provide an opportunity not only to compare the genomic organization of their β-keratins, but to study their molecular evolution in archosaurians.

**Results:**

The subfamilies (claw, feather, feather-like, and scale) of β-keratin genes are clustered in the same 5' to 3' order on microchromosome 25 in chicken and zebra finch, although the number of claw and feather genes differs between the species. Molecular phylogenies show that the monophyletic scale genes are the basal group within birds and that the monophyletic avian claw genes form the basal group to all feather and feather-like genes. Both species have a number of feather clades on microchromosome 27 that form monophyletic groups. An additional monophyletic cluster of feather genes exist on macrochromosome 2 for each species. Expression sequence tag analysis for the chicken demonstrates that all feather β-keratin clades are expressed.

**Conclusions:**

Similarity in the overall genomic organization of β-keratins in Galliformes and Passeriformes suggests similar organization in all Neognathae birds, and perhaps in the ancestral lineages leading to modern birds, such as the paravian *Anchiornis huxleyi*. Phylogenetic analyses demonstrate that evolution of archosaurian epidermal appendages in the lineage leading to birds was accompanied by duplication and divergence of an ancestral β-keratin gene cluster. As morphological diversification of epidermal appendages occurred and the β-keratin multigene family expanded, novel β-keratin genes were selected for novel functions within appendages such as feathers.

## Background

The skin of terrestrial vertebrates evolved to prevent water loss and to provide a barrier between the organism and its environment [1]. In reptiles and birds, skin appendages such as claws, scales, beaks and feathers develop, and provide novel functions. These diverse epidermal structures are composed of beta (β) keratins, whose genes have been isolated from all major groups of reptiles including squamates, crocodilians, and chelonians [2-6]. To date β-keratin sequences are known for three crocodilian species, which are highly similar to avian β-keratins [4,6]. Initial analysis of the chicken genome demonstrated that there are ~150 avian β-keratins with a tandem array of 30 being located on microchromosome 27 [7]. Recently, the feather subfamily of β-keratins has been located on multiple chromosomes in the chicken genome, yet the claw, feather-like, and scale β-keratins are restricted to microchromosome 25 [8]. Glenn et al. [9] isolated multiple copies of feather β-keratins from eight orders of the class aves, but they were unable to amplify sequences in the Passeriforme order, which makes up over fifty percent of all living birds.

Phenotypically the chicken has a larger body size and longer life span than the zebra finch. Furthermore altricial birds like the zebra finch, hatch naked, blind, and nearly helpless and are dependent on their parents for survival. In contrast precocial birds, such as the chicken are generally well-developed, fully feathered and need little parental care [10].

### Expression of Chicken β-keratins

In the chicken four subfamilies (claw, feather, feather-like and scale) of β-keratin genes have been named in accordance with tissue specific expression and sequence heterogeneity [11-13]. However, during development of the epidermis and its appendages more than one subfamily may be expressed in a specific tissue (Table 1). For example, the feather-like gene is not only expressed in feathers, but also in embryonic scales, and claw genes are expressed in embryonic feathers [12,13]. Furthermore the scaleless (*sc/sc*) mutant chicken, which does not undergo scale and feather development, expresses β-keratins from all four subfamilies in its embryonic epidermis [14]. This embryonic epidermis is generated by the initial stem cell population of the embryonic ectoderm [15].

**Table 1:** Expression of β-keratin Sequences in Chicken Tissue:

Sequence annotation	Cultured Newborn chick Keratinocytes	Embryonic chick claw tissue	Embryonic chick scale tissue	Embryonic chick feather	Adult chick feather	Embryonic chick beak
Claw	++	++++	++	+	unknown	+++

Feather	++	+*	+*	++++	++++	+*

Feather-like	unknown	none	+	++	++	none

Scale	++	+*	++++	+	unknown	+*

Keratinocyte	++	unknown	unknown	unknown	unknown	unknown

The sequence annotation for each type of β-keratin gene (claw, feather, feather-like, scale, and keratinocyte) is listed in the left column. The expression level is indicated as plus (+) signs, with more plus signs signifying higher levels of expression. The tissue source and developmental stage is designate at the top of each column. An asterisk indicates that a positive reaction was seen with antiserum for that specific β-keratin sequence [11-14,64].

As appendage morphogenesis and epidermal differentiation progress in normal birds, new epidermal stem cell lineages (germinative basal cell populations) differentiate and the expression of the β-keratin subfamilies becomes more restricted to specific appendages [15,16].

Interestingly, the four subfamilies of β-keratin genes form a cluster on microchromosome 25 (GGA25), and form monophyletic groups [8]. In the case of the chicken, members of the feather subfamily are located on 6 different chromosomes in addition to GGA25 [8]. Although we have a genomic map of the β-keratins in the chicken, we are far from understanding how the individual genes in these specific β-keratin subfamilies are utilized to build all the epidermal appendages such as the beak, spur, egg tooth, lingual nail or the numerous types of feathers [17-19].

In addition to the four subfamilies of β-keratins, two novel β-keratins have also been identified in separate experimental approaches; one from serially cultured chicken keratinocytes [20] and another from jun-transformed quail fibroblasts [21].

### Structure of the β-keratin Protein

**Figure 1:**
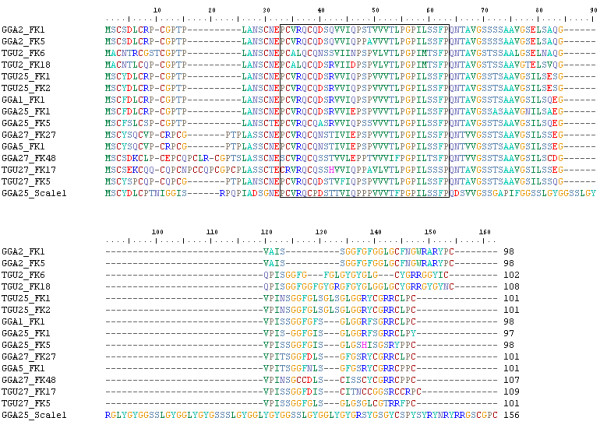
**Amino Acid Alignment of β-keratins showing the 32aa Filament Segment of Feathers**: Alignment of the two most diverged feather β-keratins from each chromosome of *G. gallus *and *T. guttata*. Annotation of the sequences includes the three letter abbreviation of the species, the chromosome number, type of β-keratin, and the number indicating position in the 5' to 3' direction on each chromosome. The 31 amino acids in the box comprise those described by Fraser and Parry [22] as the 32-residue segment constituting the filament framework of feather β-keratins. Both the *G. gallus *and *T. guttata *β-keratins possess a deletion in position 3 of the 32-residue segment.

Feather β-keratins are fibrous proteins that have four repeating units of two β-sheets that form a helical structure. This structure is surrounded by a matrix that makes up the filament-matrix texture that is seen in the structure of feathers. Fraser and Parry [22] found through X-ray diffraction studies that a 32 amino acid segment, of the total 97 amino acids that comprise the feather β-keratin coding region, makes up the 2-3 nm filament and that the remaining residues comprise the matrix (Figure 1). This is in contrast to the alpha (α)-keratins (intermediate filaments), which have a coiled coil α-helix structure and have associated amorphous proteins [23]. Based on sequence similarity, this 32 amino acid residue has also been identified in the β-keratins of scales and claws from reptiles and birds in addition to the chicken, suggesting that it is an important region and should be under intense purifying selection.

### Gene duplication

Evolution of multigene families is believed to occur through gene duplication. Duplication is relatively common and occurs by several methods, including unequal crossing over, gene conversion, and transposition by genomic elements. [24,25]. Unequal crossing over and gene conversion are often linked to tandem duplication, which results in arrays of similar regions of DNA. Transpositions are the result of transposable elements and can result in tandem duplication or the duplication of genes to other loci in a genome or species [25-28]. Lynch et al. [24] points out three possible outcomes of gene duplication: non-functionalization, in which one gene is silenced; neo-functionalization, where one of the copies acquires a new function; and sub-functionalization, in which case both copies become partially compromised [29-31]. It has been proposed that the feather β-keratin subfamily evolved from the scale β-keratin subfamily through a deletion event followed by gene duplication [11,28], but other authors suggest that the feather genes are basal to the avian scale genes [4].

### Genomic Organization of β-keratins in Gallus gallus domesticus

**Figure 2:**
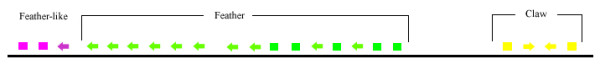
**The Genomic Organization of the β-keratin Subfamilies of *Gallus gallus domesticus***: Reproduction of the ~100 kb region containing the β-keratin gene cluster of *Gallus gallus domesticus *from Presland et al [12]. Arrows indicate the transcriptional orientation of the coding regions, where known. The boxes represent coding regions of unknown transcriptional orientation. The three β-keratin subfamilies (feather-like, feather, and claw) are labeled.

In the White Leghorn chicken (*Gallus gallus domesticus*), a genomic region spanning ~100 kb containing the claw, feather, and feather-like subfamilies of the β-keratin multigene family was described by Presland et al [12] (Figure 2). This region was reconstructed from a chicken cosmid library and a feather probe was used to identify β-keratins. The library revealed 4 claw β-keratins, 18 feather β-keratins, and 3 feather-like β-keratins in this 5' to 3' order, respectively. The scale β-keratins were not mapped in that study.

The genomes of *Gallus gallus *and *Taeniopygia guttata *have been sequenced to a 6.6 X coverage and a 5.5 X coverage respectfully [7,32]. The *G. gallus *genome was the first avian genome to be sequenced and recently the 2.1 build has been released. With information about β-keratin genes in the chicken [8,12] and the recent publication of the zebra finch genome, an in depth comparison of avian β-keratin genes and their molecular evolution is now possible in these species which differ phenotypically and are separated by ~87-111mya [33-36].

## Results

We have identified a total of 111 complete β-keratin gene sequences (coding regions) in the *G. gallus *genome [7,8], which are distributed on three macrochromosomes (GGA1, 2 and 5), one intermediate chromosome (GGA6), two microchromosomes (GGA25, and 27), and "chromosome unknown" (GGA_Un) (see Table 2) [37,38]. In the genome of *T. guttata *a total of 108 β-keratin genes are located on one macrochromosome (TGU2), two microchromosomes (TGU25 and 27), and "chromosome unknown" (TGU_Un). We also identified the number of probable genes and pseudogenes in each subfamily of β-keratin found in both genomes. These probable genes contain reasonable stop and start codons, no in-frame stop codons, no frame shift mutations, or regions of unknown genomic sequence, and they meet stringent E-values (Table 2, 3).

**Table 2:** Comparison of the Type and Number of β-keratin Genes Found on Each Chromosome.

Locus	Claw		Feather		Feather-like		Scale		Keratinocyte		BKJ	
	
	**Prob**.	Pseudo	**Prob**.	Pseudo	**Prob**.	Pseudo	**Prob**.	Pseudo	**Prob**.	Pseudo	**Prob**.	Pseudo
GGA1	0	0	1	0	0	0	0	0	0	0	0	0

GGA2	0	0	5	2	0	0	0	0	0	0	0	0

GGA5	0	0	1	0	0	0	0	0	0	0	0	0

GGA6	0	0	0	0	0	0	0	0	0	0	3	0

GGA25	8	3	15	1	4	0	4	13	1	8	0	0

GGA27	0	0	61	6	0	0	0	0	0	0	0	0

GGA_Un	0	3	8	2	0	0	0	0	0	0	0	4

TGU2	0	0	22	5	0	0	0	0	0	0	0	0

TGU25	1	6	2	0	3	0	4	7	1	10	0	0

TGU27	0	0	41	2	0	0	0	0	0	0	0	0

TGU_Un	0	2	34	6	1	1	0	2	0	1	0	4

Comparison of the number of β-keratin coding regions found on each chromosome of both *G. gallus *and *T. guttata*. The identification of each probable (Prob.) gene is based on highest similarity to the cDNA nucleotide sequences listed in Table 3. All BLAST hits that failed to meet the stringent standards were determined to be pseudogenes (pseudo) (see **Methods **for details).

**Table 3:** Query Sequences used to Perform BLAST Searches of the *G. gallus *and *T. guttata *genomes.

Feature	Lowest E-Value (*G. gallus*/*T. guttata*)	Cut-off E-Value (*G. gallus*/*T. guttata*)	Highest BLAST Score (*G. gallus*/*T. guttata*)	Cut-off BLAST score (*G. gallus/T. guttata*)	GI # or publication
Feather β-Keratin Coding (FK) [A,B,C,D]	1.00e-162/ 7.00e-70	3.00e-95/2.00e-60	565/258	342/226	GI:62929 (A) GI:62930 (B) GI: 126165215 (C) GI:62931 (D)

Feather-Like Coding (FL)	1.00e-155/5.00e-113	2.00e-93/6.00e-68	542/401	337/251	Presland et al [11]

Claw β-keratin Coding	6.00e-151/4.00e-53	4.00e-125/4.00e-53	528/202	442/202	GI:211431

Scale β-keratin Coding	5.00e-95/9.00e-57	1.00e-75/9.00e-54	339/211	274/201	GI:63548

Keratinocyte	0.0/NA	0.0/NA	743/NA	743/NA	GI:154800479

Beak β-keratin	NA/NA	NA/NA	NA/NA	NA/NA	GI:29570104

β-keratin in jun-transformed cells (BKJ)	1.00e-143/NA	1.00e-143/NA	511/NA	511/NA	GI:1016678

Query sequences used in BLAST searches and the NCBI GI number or publication where these sequences can be located. The lowest and highest (cut-off) E-value, the highest and lowest (cut-off) BLAST score for identification of β-keratin sequences on GGA25 and TGU25 are listed. These sequences were also used to search the entire genomes of both *G. gallus *and *T. guttata *using an E-value cut-off of 1e-10 for those searches involving additional feather β-keratins [7,8].

### Molecular Phylogeny

**Figure 3:**
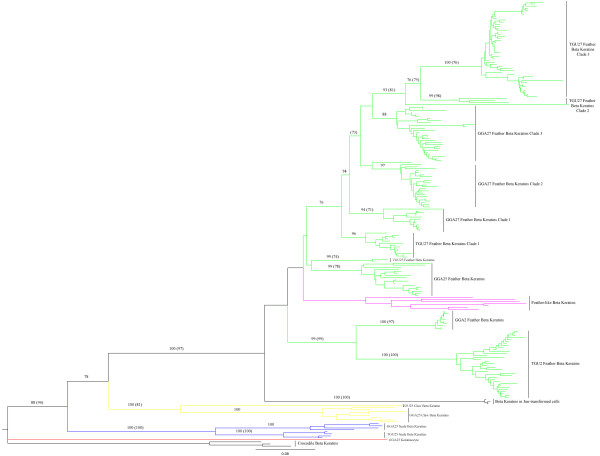
**Phylogenetic Tree Reconstruction of all β-keratin Sequences Located on the Genomes of both *G. gallus *and *T. guttata***: Three nile crocodile (*Crocodylus niloticus*) β-keratin genes are constrained as the out group [4] with all 219 β-keratin genes found in the two avian genomes. The BS (bootstrap values) from the Neighbor-Joining method are listed for each major branch when they are above 70 percent. The bootstrap values from the Maximum Likelihood method, are listed in parenthesis for each major branch when they are above 70 percent (see **Methods**) [56,60]. All individual taxa names have been removed and instead only the names of major groups are displayed. The subfamilies are colored with the following scheme: GGA25_Keratinocyte = red, scale β-keratin genes = blue, claw β-keratin genes = yellow, feather β-keratin genes = green and feather-like β-keratin genes = magenta. Please refer to Additional File 5, Figure S2 to view all bootstrap values and taxa labels for the Neighbor-Joining phylogeny and Additional File 6, Figure S3 to view all bootstrap values and taxa labels for the Maximum Likelihood phylogeny.

The molecular phylogeny of 219 probable avian β-keratin sequences, using crocodilian β-keratin genes as the outgroup demonstrates that the avian scale β-keratins are the basal group in birds (Figure 3). The β-keratin from cultured chicken keratinocytes, found only on GGA_25, is basal to the avian scale; however its expression in normal tissues *in vivo *has not been shown. The avian claw genes are basal to the feather genes, and the claw and scale genes are monophyletic, and form sister groups between chicken and zebra finch.

The feather genes on macrochromosome 2 form a monophyletic group and form sister groups between the two species. Twelve feather genes from TGU_Un sorted out with the monophyletic group of TGU2 and 5 feather genes from GGA_Un sorted out with the monophyletic group of GGA2. The β-keratin genes on GGA6, identified as being similar to those isolated from jun-transformed fibroblasts form a paraphyletic group with the monophyletic group of feather genes on macrochromosome 2, the monophyletic group of feather genes on GGA25 (which includes the feather gene on GGA1 and one feather gene from GGA_Un), the monophyletic group of feather genes on TGU25, and the feather-like genes on TGU25 and GGA25. Except for the feather-like genes, all sister groups are monophyletic. These sister groups and the feather-like genes are basal to all of the feather genes on microchromosome 27 including those from "chromosome unknown". TGU27_clade 1 is basal to 2 other clades of TGU27 and 3 clades of GGA27 feather β-keratins (Figure 3).

#### Characterization and Identification of β-keratins on Microchromosome 25

**Figure 4:**
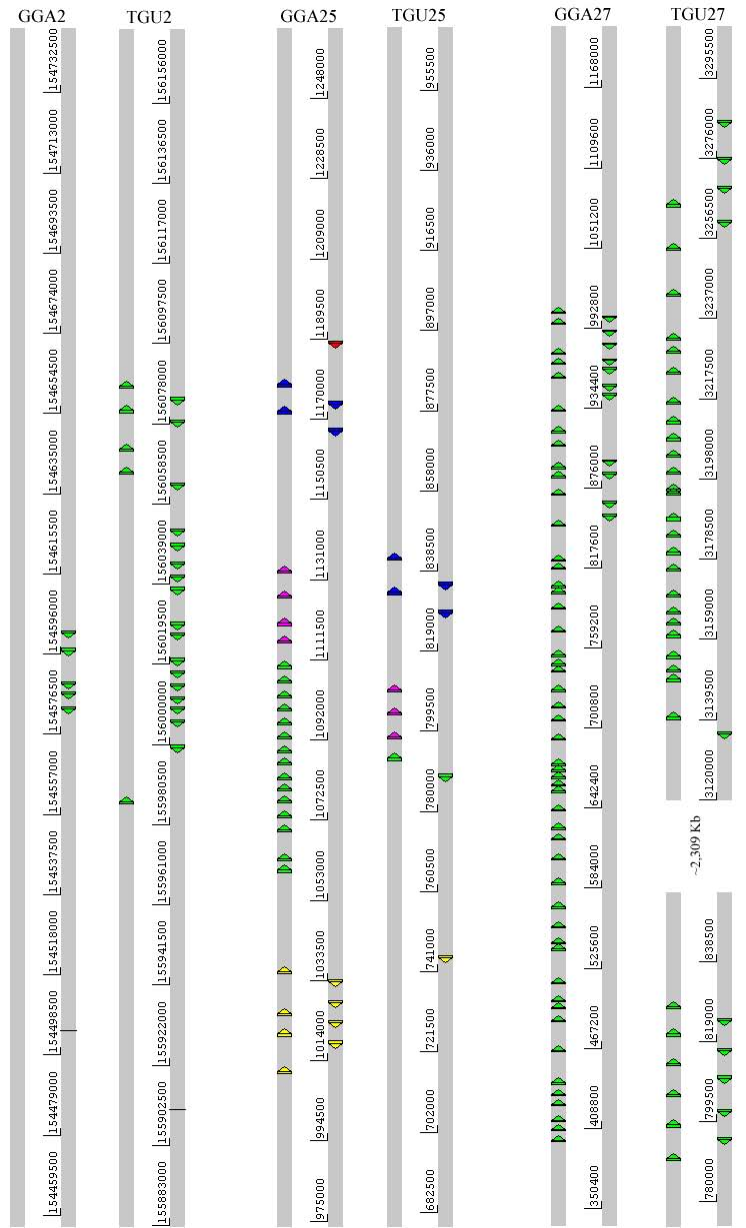
**Comparison of the β-keratin Genomic Regions in the *G. gallus *and *T. guttata *Genomes**: This figure was constructed using the Artemis program [53] and shows both the positive and negative strands of all genomic loci containing more than three β-keratin genes. Each chromosomal region is annotated above by the three letter species name and the chromosome number. The β-keratin coding regions are colored with the following scheme: scale β-keratin genes = blue, claw β-keratin genes = yellow, feather β-keratin genes = green and feather-like β-keratin genes = magenta. The arrow and strand indicate the directional orientation of each β-keratin gene. Base ranges for each chromosome are listed between the two strands. All labels are based on NCBI BLAST results with cDNA from previous expression studies [8,52,53].

Microchromosome 25 of both species displays all four subfamilies. The gene order seen on microchromosome 25, in a 5' to 3' direction, is claw, feather, feather-like, and scale (Figure 4). The overall size of the β-keratin cluster on microchromosome 25 varied between the two species with a base pair range of ~178 kb and ~120 kb for *G. gallus *and *T. guttata *respectfully. The chromosomal position of the β-keratin cluster differs for each species with the region on GGA25 being ~307 kb upstream from the β-keratin genomic cluster on TGU25.

#### β-keratin from cultured keratinocytes

One β-keratin from cultured keratinocytes was identified in the genome of *G. gallus*. This one coding region was found ~10 kb downstream of the scale β-keratins on the negative strand of GGA25 (Figure 4). This β-keratin sequence has very low overall similarity to other avian β-keratins, but of particular interest is an approximate 49 amino acid region beginning at the twentieth amino acid of the keratinocyte β-keratin coding region that shows high similarity with other β-keratin family members. This region includes the 32 amino acids described by Fraser and Parry [22] that make up the filament framework of β-keratins. Several strong hits resulted from a BLAST search of the zebra finch genome, but these hits either lacked a reasonable stop codon or contained in frame stop codons.

#### Scale β-Keratins

The genomic location, orientation and number of the scale β-keratins on chromosome 25 of *G. gallus *and *T. guttata *share striking similarities. Both species contain four scale β-keratin coding regions, which are downstream of the feather-like β-keratins on chromosome 25 (Figure 4). Both have similar orientations of the scale β-keratins, with a negative and positive strand alternating pattern. The distance between the feather-like β-keratins and the scale β-keratins is 34,176 and 19,033 bps for *G. gallus *and *T. guttata*, respectively. The total distance covered by scale β-keratins on their respective chromosome 25 is shorter on *G*. *gallus *having an 11,260 bp range and *T. guttata *having a 13,231 bp range. A ~6.5 kb center to center distance is seen between both GGA25_scale1 and 3; and GGA25_scale2 and 4, which are the coding regions found on the same strands. *T. guttata *lacks the conserved distance between sequences on the same strand, with TGU25_scale1 and 3 having ~7 kb and TGU25_scale2 and 4 having ~8 kb center to center distance.

#### Claw β-keratins

While Presland et al. [12] found four claw genes in *G. gallus domesticus*; we have identified 8 complete genes in *G. gallus*. Both lineages display positive and negative strand orientation. Regular spacing of less than 1 kb is observed for the claw β-keratins in the Galliformes. However, in *G. gallus *we removed three claw genes from the final dataset because they contained regions of unknown sequence or frame shift mutations and might be pseudogenes. Two of these potential pseudogenes are located between GGA25_Claw1 and 2 with a negative and positive strand orientation, and a third is located on the positive strand between GGA25_Claw6 and 7. The presence of these pseudogenes supports the view that the claw β-keratin genes have undergone duplication events [39].

Significant differences are seen between the claw β-keratin genes of *G. gallus *and *T. guttata*. GGA25 contains eight claw genes while TGU25 only has one (Figure 4). The sequence similarity of TGU25_Claw1 to the claw genes found in the chicken is rather low with only 49-55% identity observed from an amino acid alignment (data not shown).

#### β-keratin in jun-transformed cells (BKJ)

The only β-keratins found on GGA6 were found to be highly similar to the β-keratin isolated from quail (*Coturnix japonica*) fibroblast cells that were jun-transformed [21]. No significant blastn results were obtained from a search of the *T. guttata *genome, although the highest similarity was to TGU25_FL2. The coding regions found in the *G. gallus *genome are tandemly arrayed on chromosome 6 with unequal spacing over a ~11.7 kb range and all having a length of 109 amino acids, which is consistent with the cDNA of *C. japonica*. These coding regions have the highest similarity to feather-like β-keratins at 63.3% in an alignment with all subfamilies of the multigene β-keratin family (data not shown). A nucleotide alignment (data not shown) shows that the three BKJ coding regions only differ by one or two nucleotide changes and therefore have a greater than 99% identity between the three sequences.

#### Feather β-keratins on Macrochromosome 2

Chicken macrochromosome 2 has five complete coding regions spanning ~2.6 kb and in contrast TGU2 has twenty-two feather β-keratins located in a region spanning ~101 kb (Figure 4). The molecular phylogeny demonstrates that additional feather genes similar to those on macrochromosome 2 may be found on chromosome unknown of both species (Figure 3). Five feather genes on GGA_Un sort with the feather genes found on GGA2 and have not yet been placed in the current build of the chicken. Additionally, 12 feather genes from TGU_Un sort with those on TGU2 making the total number of genes on TGU2 34.

#### Feather-like β-keratins

There are 4 feather-like β-keratin genes on microchromosome 25 of *G. gallus *(Figure 4), while *G. gallus domesticus *contains three feather-like coding regions [12], as seen in *T. guttata*. Feather-like pseudogenes were found on chromosomes 7 and 10 in *G. gallus*. No additional feather-like genes were found outside of TGU25 in the zebra finch.

#### Feather β-keratins on Microchromosome 25

We found 15 feather β-keratin genes on microchromosome 25 of *G. gallus*, which is in contrast to the 18 identified by Presland et al [12] for *G. gallus domesticus*. All 15 coding regions are found on the positive strand and have equal spacing of ~3 kb in agreement with Presland et al [12] (see Figure 2, 4). A large gap is seen in the feather β-keratin region on GGA25, which is located between GGA25_FK2 and 3. A pseudogene is present here, but was excluded from our data because it contains a frame shift mutation. This type of mutation is evidence of a duplication event and in an array of genes indicates that some of these genes are the product of tandem duplication [39].

Only two feather coding regions are located on microchromosome 25 of *T. guttata *and they have a negative and positive strand orientation with approximately 3.5 kb separating the two genes (Figure 4).

#### Feather β-keratins on GGA1 and 5

Both avian genomes contain coding regions at other loci with high similarity to the feather β-keratins found on chromosome 25. All loci outside of chromosome 25 have tandem arrays with the exception of GGA1 and GGA5. Both of these loci contain one feather β-keratin coding region.

The molecular phylogeny (Figure 3) shows that the sequence on macrochromosome 1 sorts with the feather β-keratins of GGA25. Similar to the relationship of GGA1 and GGA25 is that of GGA5_FK1 with its paralogues on GGA27_clade2 (Figure 3). In fact, the single feather β-keratin sequence on GGA5 has a very high similarity to GGA27_FK35 with only one synonymous substitution (data not shown).

#### Feather β-keratins on Microchromosome 27

Both species have the largest number of β-keratins with the greatest similarity to feather keratins on microchromosome 27. A tandem array of 61 coding sequences is located on GGA27, in which 11 are found on the negative strand toward the 3' end of the cluster (Figure 4). The remaining 50 are found on the positive strand. TGU27 has two tandem arrays totaling 41 feather β-keratins. The array farthest downstream in the 3' direction has a similar orientation to that of GGA27 (Figure 4), but has a reduced number of coding regions. Unique to TGU27 is a second array found ~2,309 kb upstream, containing eleven coding regions.

### Analysis of Selection acting on the Feather β-keratins

Fraser and Parry, through X-ray diffraction studies, described the structure of the feather molecule. The central region, 32 amino acids of the total 97 amino acid protein, forms the filament with the remaining amino acids comprising the matrix (see Figure 1) [22]. We hypothesize that the filament region should be under purifying selection for the proper formation of feathers. In order to determine whether the large numbers of feather β-keratin genes found throughout the avian genome are actively transcribed for feather formation, the filament region was analyzed at the amino acid level by determining the individual d_*N*_/d_*S *_ratio.

The PAML program was used to analyze each chromosomal locus separately that consists of more than two feather genes (see **Methods **and Additional File 1, Table S1). The only significant results for high d_*N*_/d_*S *_ratios at individual amino acid sites in the filament region, using the PAML Program, were found at sites 37 and 38 of the feather genes on GGA2. However, the Likelihood Ratio Tests (LRTs) to determine the fit of the models, using four separate tests (M0 vs. M2a; M0 vs. M3; M1a vs. M2a; and M7 vs. M8), to the data did not significantly differ from the chi-squared value (see **Methods **and Additional File 2, Table S2), indicating a false positive result. This result may be due to the low number of feather genes analyzed on GGA2 (5 feather β-keratin genes).

### Expression of β-keratins

The Expressed sequence tag (EST) databases for the chicken and zebra finch were downloaded via NCBI and contained 599,999 and 91,801 sequences, respectably. Seventy chicken EST sequences were found to be highly similar to β-keratin sequences found in the genome of *G. gallus *(see Methods). These results include a total of forty-three separate chicken β-keratin genomic sequences, which consists of feather β-keratin sequences found on GGA1, 2, 25, and all clades of GGA27, and claw, scale, and keratinocyte β-keratin sequences of GGA25. Thus, genes of feather β-keratins are expressed from all major clades (Figure 3). However, the tissue sources of the chicken EST data are extremely diverse; ovaries, testes, eye, fat, spleen, breast muscle, and various other glands. The only significant result for the β-keratin sequences of *T. guttata *was to the TGU2_FK22 sequence, in which the resulting EST only covered the first 291 nucleotides of the 336 base pair sequence with 98% similarity. The majority of the *T. guttata *ESTs are from brain tissues (Additional File 3, Table S3).

### Gene Conversion

In order to investigate occurrences of gene conversion and/or unequal crossing over, the program GENECONV was used to analyze all 219 β-keratin genes in this data set (see **Methods **for details). The main questions addressed here were: are the β-keratin subfamilies (scale, claw, feather, feather-like) formed through unequal crossing over and subsequent divergence, and did gene conversion play a significant role in the homogenization of the these subfamilies in the avian genome [31]? Seven fragments in our data set were found to be statistically significant and support the view that unequal crossing over has occurred on GGA25. Five of these fragments, which are 33 nucleotides long, were found between GGA25_Claw6 and GGA25_FK1, 7, 8, 10 and 12. Two were between GGA25_FL1 andGGA25_FK6 and 9 and were 72 nucleotides long. These seven fragments demonstrate that the feather and feather-like β-keratins on GGA25 were formed through unequal crossing over. In contrast, a fragment of 146 nucleotides was found between two TGU_Un feather genes, which demonstrates homogenization of the feather β-keratins in the zebra finch.

## Discussion

### Genome Assembly

The actual number of β-keratin genes at any locus may not be correct because diploid genomes may contain mis-assembled contigs that result from allelic variation [40]. This variation is often localized to "chromosome unknown", but may occur when a second allelic copy is inserted next to a sister copy on a chromosome, which results in an apparent duplication of the gene or sequence [40]. In fact, the loci containing β-keratins are made up of several separate contigs. For example the genomic contig that contains the feather β-keratin clades of GGA27 consists of 162 separate contigs (see Table 4). Therefore conclusions in this study rely more heavily on phylogenetic results (Figure 3) than on the actual number of β-keratin genes. The EST data in combination with molecular phylogenies demonstrate that the β-keratin multigene family does consist of multiple clades of feather β-keratins found on many loci throughout the genomes of the chicken and zebra finch.

**Table 4:** Genomic Contigs that contain Clusters of β-keratins and their Composition.

**Locus**	**Genomic Contig**	**Total number of nucleotides**	**Chromosome nucleotide range**	**Number of sub-contigs**	**Estimated** **gap length (ntd)**
GGA2	NW_001471655	6,197,148	148409042-154606189	233	141,810

TGU2	NW_002198285	1,423,962	154736755-156160716	187	18,600

GGA6	NW_001471714	6,665,001	4744693-11409693	193	119,893

GGA25	NW_001471598	380,842	1009775-1390616	41	31,241

TGU25	NW_002198052	407,827	621358-1029184	80	7,900

GGA27	NW_001471611	1,465,288	1-1465288	162	85,846

TGU27_Loci_1	NW_002198174	148,626	788428-937053	14	1,300

TGU27_Loci_2	NW_002198180	666,370	3132406-3798775	68	6,700

Each genomic contig that contains at least two β-keratin sequences are listed according to their NCBI GI number. For each genomic contig the total number of nucleotides, the range of nucleotides that is covered on their respective chromosome, the number of contigs, and the estimated gap lengths based on linkage maps for each species is listed. In the case of zebra finch, each gap consisted of 100 unknown nucleotides.

In the case of *G. gallus *microchromosome 25, a similar number of β-keratin genes from three subfamilies (claw, feather, and feather-like) with the same genomic organization was observed in *G. gallus domesticus *by Presland et al. [12]. They [12] reconstructed this region from a chicken cosmid library using restriction enzymes to map the ~100 kb genomic fragments (see Figure 2). Comparison of this region with the region we have identified on GGA25 (from the start of GGA25_claw6 to the end of GGA25_FL3 spanning ~99 kb distance, see Figure 4) indicates that this region of the chicken genome build 2.1 is of high quality.

### Molecular phylogeny

A comparison of the trees generated by the Neighbor-Joining method (see Additional file 5) and the Maximum Likelihood method (see Additional file 6) shows that generally the Maximum Likelihood has more conservative bootstrap values. Although the Maximum Likelihood phylogeny shows that the claw and scale β-keratin genes are monophyletic it does not support the paraphyletic grouping by species. Furthermore, the bootstrap support for TGU27 clade 1 being monophyletic and the basal group for the other feather β-keratin genes on microchromosome 27 is very weak. GGA27 clade 2 and 3 also lack strong bootstrap support for their monophyly. Overall the trees are very similar, but the low bootstrap support from Maximum Likelihood analysis may reflect the high similarity (indicating fewer segregating sites) within the subfamilies of β-keratins and the robustness of this method.

### Expression of β-keratins and Phenotypic Variation between the Chicken and Zebra Finch

Studies of protein expression using two-dimensional gel electrophoresis have demonstrated that overlap exists between the proteins expressed by the claw, egg tooth, beak and scale from 19-20 day chick embryos (Additional File 4, Figure S1). These tissues express different levels of β-keratins. For example, the scale and beak express at least 7 different β-keratins and several phosphorylated β-keratins, while the egg tooth and claw have significantly reduced levels of two of these β-keratins and two phosphorylated β-keratins [13]. The beak, scale and claw tissues display very similar expression patterns for their β-keratins on two-dimensional gels (Additional File 4, Figure S1). These data suggest that not only do the scale epidermal cells express scale β-keratin genes but they also express β-keratin genes from other subfamilies such as claw. Furthermore, some β-keratin genes are expressed at the same level in multiple epidermal appendages (claw, egg tooth, beak, and scale), while others are expressed at significantly reduced levels [13]. Interestingly, we have found that the gene isolated from embryonic chicken beak (Wu et al, unpublished results [41]) has 97% identity with GGA25_Claw6 (results not shown) further demonstrating that the beak epidermis expresses the claw subfamily of β-keratins.

No information is available on the expression of β-keratins in the claws or beaks of passeriformes. It may be that the single claw gene (TGU25_Claw1) in the zebra finch genome is expressed in both structures. However, a recent study of claw development in the zebra finch suggests that the zebra finch claw is a modified scale [42]. Perhaps the zebra finch claw epidermis uses β-keratins from the scale subfamily and/or other subfamilies for its cornification. Furthermore since the beaks of chickens express both scale and claw genes (Table 1), perhaps the beaks of passeriformes express mainly scale β-keratins [19].

Passeriformes are altricial birds [10], and are mostly naked when they hatch. In the zebra finch a few embryonic feather filaments are scattered over the body of the hatchling [43]. Assuming that the number of genes on microchromosome 25 is correct for the chicken and zebra finch, the differences in the number of feather β-keratin genes on microchromosome 25 for these two species may be related to the altricial nature of passeriformes. Studies do demonstrate that the feather β-keratin genes on GGA25 are expressed in developing feathers [11,12]. If the feather genes on microchromosome 25 are used mainly to produce the structural proteins for the embryonic down feathers, then the demand for multiple copies of feather genes [44-46] may be relaxed in altricial birds.

### Recombination

The results of the gene conversion test (See **Results**) suggest that the feather genes on microchromosome 25 arose through unequal crossing over from either the claw or feather-like genes or both. An alternative hypothesis is that the claw genes, through unequal crossing over, gave rise to the feather genes which in turn gave rise to the feather-like genes found on GGA25. These results indicate that many episodes of recombination have occurred on GGA25 and that gene conversion may be very rare among β-keratin sequences. In fact, the only results that indicate gene conversion are between two feather β-keratin genes found on TGU_Un.

The high number of genes found for the clades on microchromosome 27 in both species may be due to the high rate of recombination seen on avian microchromosomes. The negative correlation between recombination and chromosome size, results in a higher gene density on microchromosomes [7,37]. The high number of feather β-keratins on TGU2 may relate to the high rate of recombination that occurs toward the ends of the macrochromosomes in the zebra finch genome [47].

### Implications for Evolution of Avian Genomes

**Figure 5:**
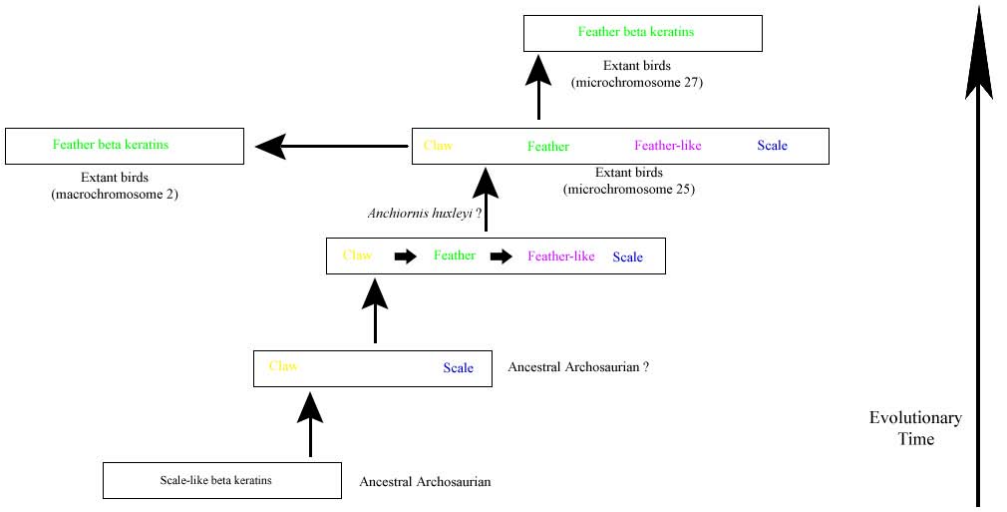
**Proposed Evolution of the β-keratin Genomic Region**. This figure illustrates a proposed scenario for the evolution of the β-keratin subfamilies in extant birds from their archosaurian ancestor. The vertical arrows indicate evolutionary time and the horizontal arrows in the boxes indicate possible unequal crossing over events. The subfamilies are colored with the following scheme: scale β-keratin genes = blue, claw β-keratin genes = yellow, feather β-keratin genes = green and feather-like β-keratin genes = magenta.

Recently, Hu et al. [48] described a small, crow-sized theropod, *Anchiornis huxleyi*, dated to ~155mya, with long pennaceous feathers on its forelimbs and hindlimbs. They point out that large pennaceous feathers located on the hind limb is a feature known for the basal members of the three major paravian groups [see also [49]]. Hu et al. [48] further proposes that feathering of the foot was a critical step in the evolution of birds [see [50]]. Since numerous studies demonstrate that adult feathers are made of feather β-keratins [9,11,12], it is reasonable to assume that the feather and/or feather-like β-keratins were present in the pennaceous feathers on *Anchiornis *and other Paraves. Considered in light of our phylogenetic analyses of the β-keratin subfamilies, the presence of pennaceous feathers on *Anchiornis *supports the view that a β-keratin multigene cluster similar to that seen on microchromosome 25 in today's birds may have existed in archosaurians as early as the Middle Jurassic [48] (Figure 5).

## Conclusion

Our results suggest the following scenario for the evolution of the β-keratin gene family (Figure 5). The genome of early archosaurians contained a cluster of β-keratin genes, closely related to the scale β-keratin genes seen in today's crocodilians and birds [4,6,8,51]. Duplication and diversification lead to the subfamily known as claw, which provided additional building blocks for the evolution of archosaurian appendages; i. e., claws, beaks, spurs, etc [17-19]. In fact, members of both the scale and claw subfamilies of β-keratin are present in developing claws, beaks, scales, and even feathers of birds (Table 1 and Additional File 4, Figure S1) [11,12,19]. As the development and morphogenesis of the epidermal appendages diversified further, recombination in the β-keratin gene cluster provided the raw material for the evolution of new β-keratin genes, such as feather and feather-like, which would eventually provide the structural proteins for appendages with novel functions, such as the feather. In fact, our molecular phylogenies demonstrate that the avian claw genes evolved from the scale genes, and form a basal group to the feather-like and feather genes (Figure 3 and 5).

## Methods

### Identification and localization of the β-keratin multigene family

The β-keratin nucleotide sequences, amino acid sequences, and unique features associated with the β-keratin genes were obtained from NCBI and published sources [8]. All GI numbers or references (without GI numbers) for β-keratins are listed in Table 3. The NCBI Basic Local Alignment Search Tool (BLAST) was used to search the 2.1 build of *G. gallus *and the 1.1 build of *T. guttata *[52]. The genomic data was downloaded via the NCBI ftp site. We used Artemis 8.1 [53] to view the β-keratin genes graphically.

To identify the genomic region in *G. gallus *that contained the claw, feather, and feather-like β-keratins described by Presland et al [12] we used very strict E-values and BLAST scores. This strict use of identity was also applied to all the unique features associated with the β-keratins, including the scale β-keratins and the β-keratin from cultured keratinocytes. Table 3 identifies the highest and lowest E-value and BLAST score used for each feature of the genomic region identified by Presland et al [12] for *G. gallus *[8] and for the β-keratin in jun-transformed cells. The sequences used to search *T. guttata *were identical to those used for *G. gallus *(Table 3).

In addition to the cluster of β-keratins identified by Presland et al [12], preliminary analysis of the genomes of both *G. gallus and T. guttata *revealed additional genomic loci (including chromosome unknown of both the chicken and zebra finch) containing feather or feather-like β-keratins. To identify and classify these genes fully, more lenient parameters were used: an E-value cut-off of 1e-10, reasonable stop codons and start codons, no in-frame stop codons, no frame shift mutations, and no regions of unknown genomic sequence (see Table 2).

All sequences in this study were obtained from the genomic sequences of *G. gallus *and *T. guttata *and use a simple annotation pattern. Since all data presented in this paper is from the genomic sequences of *G. gallus *and *T. guttata*, the numbering of the β-keratins will follow a 5' to 3' pattern. This annotation also includes the species (abbreviated as GGA or TGU), chromosome number and β-keratin subfamily (feather = FK, feather-like = FL, claw = Claw, or scale = Scale and β-keratin from cultured keratinocytes = keratinocyte). For example, the claw sequence which is found at the 5' end of the cluster on the positive strand, located on microchromosome 25 of *G. gallus *is annotated as GGA25_claw1 (See Additional File 1 Table S1).

### Expressed Sequence Tag Analysis

Expressed sequence tag (EST) databases for both avian species were downloaded via NCBI and contained 599,999 and 91,801 sequences for chicken and zebra finch, respectably. The β-keratin databases for chicken and zebra finch were used as queries for blastn searches of the EST databases. An E-value cutoff of 1e-160 was used for G. *gallus *β-keratin sequences and an E-value cutoff of 1e-150 was used for the *T. guttata *β-keratin sequences. The GI number for the EST, the range of the nucleotides corresponding to the β-keratin sequence, the range of the 5' and 3' EST base pairs, and the EST tissue collection information is listed in Additional File 3, Table S3.

### Phylogenetic Analysis

Alignments were accomplished using the program CLUSTAL W Multiple Sequence Alignment Program [54] and PAL2NAL [55]. All default parameters were used in PAL2NAL and CLUSTAL W with the option SLOW/ACCURATE selected for CLUSTAL W alignments. Visual inspection confirmed an adequate alignment. Phylogenetic and molecular evolutionary analyses were conducted using MEGA version 4 [56].

Tree reconstruction was done using a total of 222 taxa, which included all probable feather β-keratins (located on GGA1, GGA2, TGU2, GGA5, GGA27, TGU27, GGA_Un and TGU_Un) and all probable β-keratins on GGA6 and chromosome 25 of both avian species (see Table 2). As an outgroup for tree reconstruction, three β-keratin nucleotide coding sequences were used from *Crocodylus niloticus *(Nile crocodile) and were obtained via NCBI and have the following Genbank numbers: 215541571, 215541573 and 187942180[4]. The Modeltest program, using the Akaike Information Criterion (AIC), selected the generalized time reversible evolutionary model with gamma distributed rate heterogeneity and a proportion of invariant sites as the best fit model for these taxa [57]. Using this suggested evolutionary model, tree reconstruction was accomplished using both distance based (Neighbor-Joining) and character based (Maximum Likelihood) methods.

MEGA version 4 [56] was used for Neighbor-Joining tree reconstruction, which was accomplished with 1000 bootstrap replicates, pairwise deletion, all codon positions selected, the LogDet evolutionary model, substitutions including transitions and transversions, and a heterogeneous pattern among lineages [58,59]. The nile crocodile sequences was chosen as the outgroup. The resulting evolutionary tree was used for figure construction (Additional file 5).

The RA×ML 7.0.3 edition was used for Maximum Likelihood analyses. 1000 bootstrap replicates was accomplished using the GTR+I+G model (GTRGAMMAI) with *C. niloticus *chosen as the outgroup. The first run was done using the -f i option, which performs a really thorough standard bootstrap procedure (1000 bootstrap replicates). A second run was done with -f d option, which performs a fast rapid hill-climbing algorithm. Bootstrap values from the first run were added to the resulting tree of the second run (Additional file 6) [60].

The large scale duplication seen among the feather β-keratin sequences were analyzed to determine amino acid sites under varying selective pressures using the PAML 4.0 package, in which six models of evolution were used [61]. Models tested were: M0 (one ratio), M1a (nearly neutral), M2a (positive selection), M3 (discrete), M7 (beta) and M8 (beta + ω). Three models allow for the possibility of positive selection, M2a, M3, and M8, which is the d_*N*_/d_*S *_ratio of ω being greater than 1. To test the fit of these models to the data and therefore the occurrence of a false positive, likelihood ratio tests (LRTs) were used. LRTs were only used when a model (M2a, M3, or M8) suggested a high d_*N*_/d_*S *_ratio for amino acid sites. These three models were tested against a null model, using four separate tests (M0 vs. M2a; M0 vs. M3; M1a vs. M2a; and M7 vs. M8), by comparing (2*l*n Δ*l*) against the χ^2^-distribution, with the degrees of freedom equal to the number of parameters between models. Each major locus that contained more than two feather β-keratins were analyzed separately (TGU2, GGA2, GGA25, GGA27 and TGU27). This allowed for the possibility that each chromosome/locus of each species (GGA and TGU) has evolved independent or temporally separate and would then be under different selective pressures. The PAL2NAL alignment tool was used to convert a nucleotide alignment into a codon alignment for input into the PAML program in association with the Neighbor-Joining (N-J) tree output from CLUSTAL W [54,56,62].

### Gene Conversion

To search for duplicated segments shared between pairs of genes, by mechanisms such as gene conversion and/or unequal crossing over, the program GENCONV 1.81a was utilized and applied to all probable genes in the final data set (See **Identification and localization of the β-keratin multigene family **above). Global Bonferroni corrected P-values were calculated against a simulated distribution of 10,000 iterations to determine the statistical significance of a shared observed fragment from a pair of sequences. To be considered a significant result the p-value must be lower than 0.01 [31,63].

## Authors' contributions

MJG generated the data as part of his Ph. D. research. RHS as the major professor assisted in preparation of the manuscript. Both authors read and approved the final manuscript.

## Supplementary Material

Additional file 1 **Annotation of β-keratins resulting from BLAST Searches of the *Gallus gallus *and *Taeniopygia guttata *Genomes**. List of all sequences used in this study that resulted from a BLAST search of the *G. gallus *and *T. guttata *genomes [7,8]. Annotation of each sequence used in this study, its position on its respective chromosome, and strand orientation is indicated by a lowercase c (complement) if it was found on the complementary strand. Additionally, the Ensembl Gene identification numbers were listed for every β-keratin sequence that was annotated by Ensembl, and was not eliminated from this study by criteria described in the Methods section. Click here for file

Additional file 2 **PAML Analysis of all Feather β-keratin Loci that Resulted in Positively Selected Sites**: The six models are listed in the first column with a brief description, and the values obtained from each analysis are listed in their respective rows. The dN/dS ratios are the average of the sum of all branches. All positively selected sites above 95% are listed, with those reaching 99% shown in bold. The Naïve Empirical Bayes (NEB) and Bayes Empirical Bayes (BEB) are shown when appropriate. The M3 model only uses the NEB analysis [61]. Additionally, the tables for the likelihood ratio test (LRT) results for each locus are included (see **Methods**). Click here for file

Additional file 3 **Expressed Sequence Tag BLAST results**. List of all β-keratin sequences in the genomes of *G. gallus *and *T. guttata *that had at least an E-value score of 1e-160 and 1e-150, respectably, for an expressed sequence tag (EST). EST database was downloaded via NCBI for each species. The GI number for each EST, region of the EST matching the coding region of the β-keratin, the 5' region outside of the coding region for each matching EST, the 3' region outside of the coding region for each matching EST, the tissue source of the EST, the developmental stage of the collected EST source and the sex are listed. Click here for file

Additional file 4 **Two-dimensional Gels of β-keratin Expression in Chick Epidermal Appendages**. Reprint of Figure 2 in Shames et al [19]. Two-dimensional gels of protein extracted from 19-20 day embryonic chick (A) scutate scale epidermis, (B) cornified beak, (C) egg tooth, (D) periderm, and (E) claw. The acidic (Ac) and basic (Ba) ends of the gel and the molecular weight markers are indicated for the second dimension. The protein spots labeled 1 and 2, 3 are scale β-keratins identified by hybrid-selection using a scale specific oligonucleotide probe [65]. Click here for file

Additional file 5 **Tree Reconstruction of all β-keratin genes found in the *Gallus gallus *and *Taeniopygia guttata *Genomes**. Neighbor-Joining tree reconstruction of the 219 β-keratin nucleotide sequences from both avian genomes and the three nile crocodile sequences as the outgroup. The subfamilies are colored with the following scheme: GGA25_Keratinocyte = red, scale β-keratin genes = blue, claw β-keratin genes = yellow, feather β-keratin genes = green and feather-like β-keratin genes = magenta. The taxa nomenclature and methodology is detailed in the Methods section. Only bootstrap values from the Neighbor-Joining method are listed. Click here for file

Additional file 6 **Tree Reconstruction of all β-keratin genes found in the *Gallus gallus *and *Taeniopygia guttata *Genomes**. Maximum Likelihood tree reconstruction of the 219 β-keratin nucleotide sequences from both avian genomes and the three nile crocodile sequences as the outgroup. The subfamilies are colored with the following scheme: GGA25_Keratinocyte = red, scale β-keratin genes = blue, claw β-keratin genes = yellow, feather β-keratin genes = green and feather-like β-keratin genes = magenta. The taxa nomenclature and methodology is detailed in the Methods section. Only bootstrap values from the Maximum Likelihood method are listed. Click here for file
