# Supplementary material for: Genomic organization and molecular phylogenies of the beta (β) keratin multigene family in the chicken (Gallus gallus) and zebra finch (Taeniopygia guttata): implications for feather evolution
Source: BMC Evol Biol. 2010 May 18;10:148. doi: 10.1186/1471-2148-10-148 (PMC2894828; doi:10.1186/1471-2148-10-148)
Supplement: Additional file 2 — PAML Analysis of all Feather β-keratin Loci that Resulted in Positively Selected Sites: The six models are listed in the first column with a brief description, and the values obtained from each analysis are listed in their respective rows. The dN/dS ratios are the average of the sum of all branches. All positively selected sites above 95% are listed, with those reaching 99% shown in bold. The Naïve Empirical Bayes (NEB) and Bayes Empirical Bayes (BEB) are shown when appropriate. The M3 model only uses the NEB analysis [61]. Additionally, the tables for the likelihood ratio test (LRT) results for each locus are included (see Methods). [file 1471-2148-10-148-S2.PDF]

Likelihood Values and Parameter Estimates for GGA25.

**Models**	***p***	***l***	**Kappa (ts/tv)**	**dN/dS**	**Estimates of parameters**	**Positively Selected Sites**
M0 (one-ratio)	1	-863.35	2.62701	0.1373	ω=0.13732	None
M1a (Nearly Neutral)	2	-851.47	2.73023	0.1435	p_0=0.91824, ω_0=0.06728<br>p_1=0.08176, ω_1=1.000	Not Allowed
M2a (Positive Selection)	4	-849.93	3.15217	0.1881	p_0=0.92372, ω_0=0.07628<br>p_1=0.06483, ω_1=1.000<br>p_2=0.01144, ω_2=4.61356	None
M3 (discrete)	5	-849.03	3.02138	0.1450	p_0=0.73450, ω_0=0.03097<br>p_1=0.24987, ω_1=0.35726<br>p_2=0.01563, ω_2=3.71894	**71**
M7 (beta)	2	-852.41	2.68632	0.1450	p=0.19098, q=1.10209	Not Allowed
M8 (beta & ω>1)	4	-849.02	3.03101	0.1704	p_0=0.98509, (p_1=0.01491)<br>p=0.35974, q=2.66377, ω=3.82638	**71**

Likelihood Values and Parameter Estimates for GGA27.

**Models**	***p***	***l***	**Kappa (ts/tv)**	**dN/dS**	**Estimates of parameters**	**Positively Selected Sites**
M0 (one-ratio)	1	-2502.91	2.7284	0.1339	ω= 0.13386	None
M1a (Nearly Neutral)	2	-2446.95	2.9577	0.2001	p_0= 0.86481, ω_0= 0.07507<br>p_1= 0.13519, ω_1=1.000	Not Allowed
M2a (Positive Selection)	4	-2446.46	3.0014	0.2203	p_0=0.86612, ω_0=0.07690<br>p_1=0.11830, ω_1=1.000<br>p_2=0.01557, ω_2=2.27184	none
M3 (discrete)	5	-2433.88	2.9135	0.1821	p_0=0.61223, ω_0=0.02796<br>p_1=0.30535, ω_1=0.24011<br>p_2=0.08242, ω_2=1.11211	**14, 41, 113**
M7 (beta)	2	-2438.64	2.8768	0.1785	p=0.32074, q=1.44843	Not Allowed
M8 (beta & ω>1)	4	-2433.78	2.9293	0.1862	p_0=0.93480, (p_1=0.06520)<br>p=0.52126, q=4.06646, ω=1.27657	NEB only: 14, **113**

Likelihood Values and Parameter Estimates for TGU27.

**Models**	***p***	***l***	**Kappa (ts/tv)**	**dN/dS**	**Estimates of parameters**	**Positively Selected Sites**
M0 (one-ratio)	1	-1616.75	1.7306	0.2283	ω= 0.22833	None
M1a (Nearly Neutral)	2	-1589.93	1.8945	0.3154	p_0= 0.76399, ω_0= 0.10390<br>p_1= 0.23601, ω_1=1.00000	Not Allowed
M2a (Positive Selection)	4	-1589.68	1.8816	0.3212	p_0= 0.75669, ω_0= 0.10293<br>p_1= 0.17399, ω_1=1.00000<br>p_2= 0.06932, ω_2= 1.00000	none
M3 (discrete)	5	-1578.23	1.8086	0.2934	p_0= 0.44627, ω_0= 0.00000<br>p_1= 0.47697, ω_1= 0.36126<br>p_2= 0.07675, ω_2= 1.57797	100, 106
M7 (beta)	2	-1581.70	1.8114	0.2715	p= 0.25581, q= 0.68526	Not Allowed

M8 (beta & ω>1)	4	-1580.55	1.8194	0.3052	p_0= 0.96984, (p_1= 0.03016) p= 0.30174, q= 0.94349, ω=2.36606	None

Likelihood Values and Parameter Estimates for GGA2.

**Models**	***p***	***l***	**Kappa (ts/tv)**	**dN/dS**	**Estimates of parameters**	**Positively Selected Sites**
M0 (one-ratio)	1	-342.45	999.0	0.36176	ω=0.36176	None
M1a (Nearly Neutral)	2	-341.21	999.0	0.1869	p_0=0.81313, ω_0=0.000<br>p_1=0.18687, ω_1=1.000	Not Allowed
M2a (Positive Selection)	4	-340.64	999.0	0.4229	p_0=0.91563, ω_0=0.000<br>p_1=0.000, ω_1=1.000<br>p_2=0.08437, ω_2=5.01284	NEB only: **5, 37, 38**
M3 (discrete)	5	-340.63	999.0	0.4229	p_0=0.82916, ω_0=0.000<br>p_1=0.08647, ω_1=0.000<br>p_2=0.08437, ω_2=5.01281	**5, 37, 38**
M7 (beta)	2	-341.22	999.0	0.200	p=0.0050, q=0.02036	Not Allowed
M8 (beta & ω>1)	4	-340.63	999.0	0.4229	p_0=0.91563, (p_1=0.08437)<br>p=0.0050, q=22.49008, ω=5.01282	NEB only: **5, 37, 38**

Likelihood Values and Parameter Estimates for TGU2.

**Models**	***p***	***l***	**Kappa (ts/tv)**	**dN/dS**	**Estimates of parameters**	**Positively Selected Sites**
M0 (one-ratio)	1	-1225.64	3.58620	0.2349	ω=0.23492	None
M1a (Nearly Neutral)	2	-1160.26	3.58594	0.2310	p_0=0.77094, ω_0=0.00256<br>p_1=0.22906, ω_1=1.00000	Not Allowed
M2a (Positive Selection)	4	-1150.09	3.88647	0.5014	p_0=0.76980, ω_0=0.00229<br>p_1=0.19057, ω_1=1.00000<br>p_2=0.03963, ω_2=7.80008	**8, 94, 98**
M3 (discrete)	5	-1150.06	3.92160	0.5158	p_0=0.77303, ω_0=0.00291<br>p_1=0.18871, ω_1=0.18871<br>p_2=0.03826, ω_2=8.17341	**6, 8, 9, 30, 32, 36, 68, 71, 72, 86, 88, 92, 94, 98, 109, 110, 111, 112**
M7 (beta)	2	-1160.42	3.57248	0.2043	p=0.00956, q=0.03242	Not Allowed
M8 (beta & ω>1)	4	-1150.08	3.88496	0.5016	p_0=0.96039, (p_1=0.03961)<br>p=0.00984, q=0.03455, ω=7.76300	**8, 94, 98**

PAML Analysis of all Feather β-keratin Loci that Resulted in Positively Selected Sites: The six models are listed in the first column with a brief description, and the values obtained from each analysis are listed in their respective rows. The dN/dS ratios are the average of the sum of all branches. All positively selected sites above 95% are listed, with those reaching 99% shown in bold. The Naïve Empirical Bayes (NEB) and Bayes Empirical Bayes (BEB) are shown when appropriate. The M3 model only uses the NEB analysis [37].

Likelihood Ratio Statistics for GGA25 among different Models given in the above Table

**Comparison**	**2Δ*l***	**df**	***p***
M0 vs. M2a	26.84	2	< 0.01
M0 vs. M3	28.64	4	< 0.01
M1a vs M2a	3.08	2	0.21
M7 vs. M8	6.78	2	0.03

Likelihood Ratio Statistics for GGA27 among different Models given in the above Table

**Comparison**	**2Δ*l***	**df**	***p***
M0 vs. M2a	112.9	2	< 0.01
M0 vs. M3	138.06	4	< 0.01
M1a vs M2a	0.98	2	0.61
M7 vs. M8	9.72	2	< 0.01

Likelihood Ratio Statistics for GGA2 among different Models given in the above Table

**Comparison**	**2Δ*l***	**df**	***p***
M0 vs. M2a	3.62	2	0.16
M0 vs. M3	3.64	4	0.46
M1a vs M2a	1.14	2	0.57
M7 vs. M8	1.18	2	0.55

Likelihood Ratio Statistics for TGU2 among different Models given in the above Table

**Comparison**	**2Δ*l***	**df**	***p***
M0 vs. M2a	151.10	2	**< 0.01**
M0 vs. M3	151.16	4	**< 0.01**
M1a vs M2a	20.34	2	**< 0.01**
M7 vs. M8	20.68	2	**< 0.01**
