# Supplementary figures and images for: Genomic organization and molecular phylogenies of the beta (β) keratin multigene family in the chicken (Gallus gallus) and zebra finch (Taeniopygia guttata): implications for feather evolution
Source: BMC Evol Biol. 2010 May 18;10:148. doi: 10.1186/1471-2148-10-148 (PMC2894828; doi:10.1186/1471-2148-10-148)

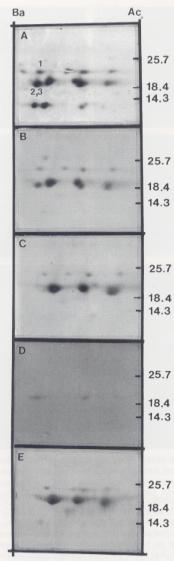

Supplement: Additional file 4 — Two-dimensional Gels of β-keratin Expression in Chick Epidermal Appendages. Reprint of Figure 2 in Shames et al [19]. Two-dimensional gels of protein extracted from 19-20 day embryonic chick (A) scutate scale epidermis, (B) cornified beak, (C) egg tooth, (D) periderm, and (E) claw. The acidic (Ac) and basic (Ba) ends of the gel and the molecular weight markers are indicated for the second dimension. The protein spots labeled 1 and 2, 3 are scale β-keratins identified by hybrid-selection using a scale specific oligonucleotide probe [65]. [file 1471-2148-10-148-S4.JPEG]

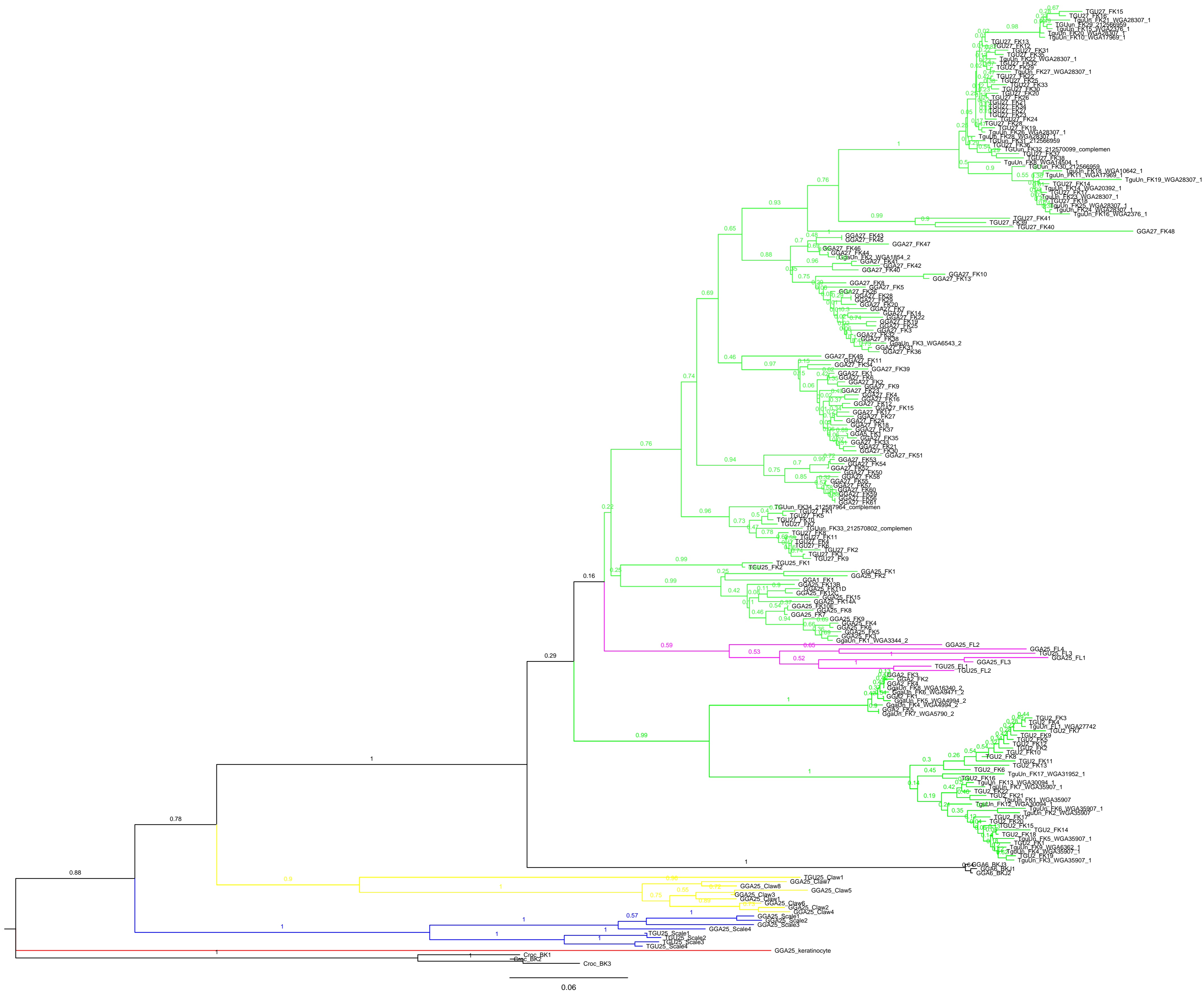

Supplement: Additional file 5 — Tree Reconstruction of all β-keratin genes found in the Gallus gallus and Taeniopygia guttata Genomes. Neighbor-Joining tree reconstruction of the 219 β-keratin nucleotide sequences from both avian genomes and the three nile crocodile sequences as the outgroup. The subfamilies are colored with the following scheme: GGA25_Keratinocyte = red, scale β-keratin genes = blue, claw β-keratin genes = yellow, feather β-keratin genes = green and feather-like β-keratin genes = magenta. The taxa nomenclature and methodology is detailed in the Methods section. Only bootstrap values from the Neighbor-Joining method are listed. [file 1471-2148-10-148-S5.PDF]
