# Supplementary material for: Genomic organization and molecular phylogenies of the beta (β) keratin multigene family in the chicken (Gallus gallus) and zebra finch (Taeniopygia guttata): implications for feather evolution
Source: BMC Evol Biol. 2010 May 18;10:148. doi: 10.1186/1471-2148-10-148 (PMC2894828; doi:10.1186/1471-2148-10-148)
Supplement: Additional file 6 — Tree Reconstruction of all β-keratin genes found in the Gallus gallus and Taeniopygia guttata Genomes. Maximum Likelihood tree reconstruction of the 219 β-keratin nucleotide sequences from both avian genomes and the three nile crocodile sequences as the outgroup. The subfamilies are colored with the following scheme: GGA25_Keratinocyte = red, scale β-keratin genes = blue, claw β-keratin genes = yellow, feather β-keratin genes = green and feather-like β-keratin genes = magenta. The taxa nomenclature and methodology is detailed in the Methods section. Only bootstrap values from the Maximum Likelihood method are listed. [file 1471-2148-10-148-S6.PDF]

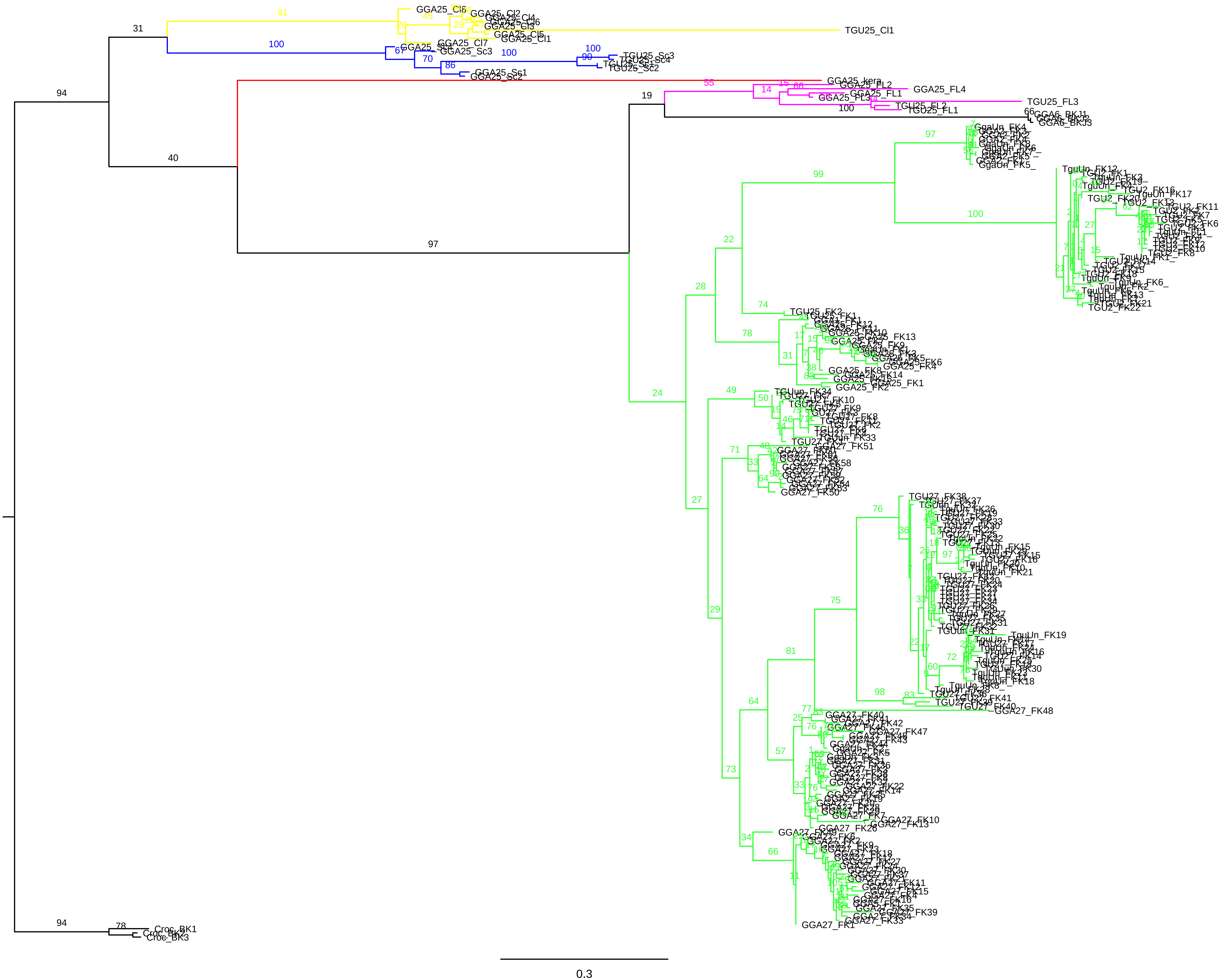

TGU25_Cl1
GGA25_Cl7
GGA25_Cl5
GGA25_Cl1
GGA25_Sc3
GGA25_Sc1
GGA25_Sc2
TGU25_Sc3
TGU25_Sc2
GGA25_kera
GGA25_FL2
GGA25_FL4
GGA25_FL1
TGU25_FL3
TGU25_FL2
TGU25_FL1
GGA6_BKJ3
GGA25_FK13
GGA25_FK6
GGA25_FK4
GGA25_FK1
GGA25_FK2
TGU25_FK2
TGU27_FK10
TGU27_FK38
TGU27_FK37
TguUn_FK19
TguUn_FK21
TGU27_FK41
GGA27_FK48
GGA27_FK40
GGA27_FK47
GGA27_FK43
GGA27_FK22
GGA27_FK14
GGA27_FK10
GGA27_FK13
GGA27_FK26
GGA27_FK39
GGA27_FK33
GGA27_FK1
GGA27_FK50
TGU2_FK11
TGU2_FK6
TGU2_FK10
TGU2_FK8
TGU2_FK21
TGU2_FK22
Croc_BK1
Croc_BK3
0.3
